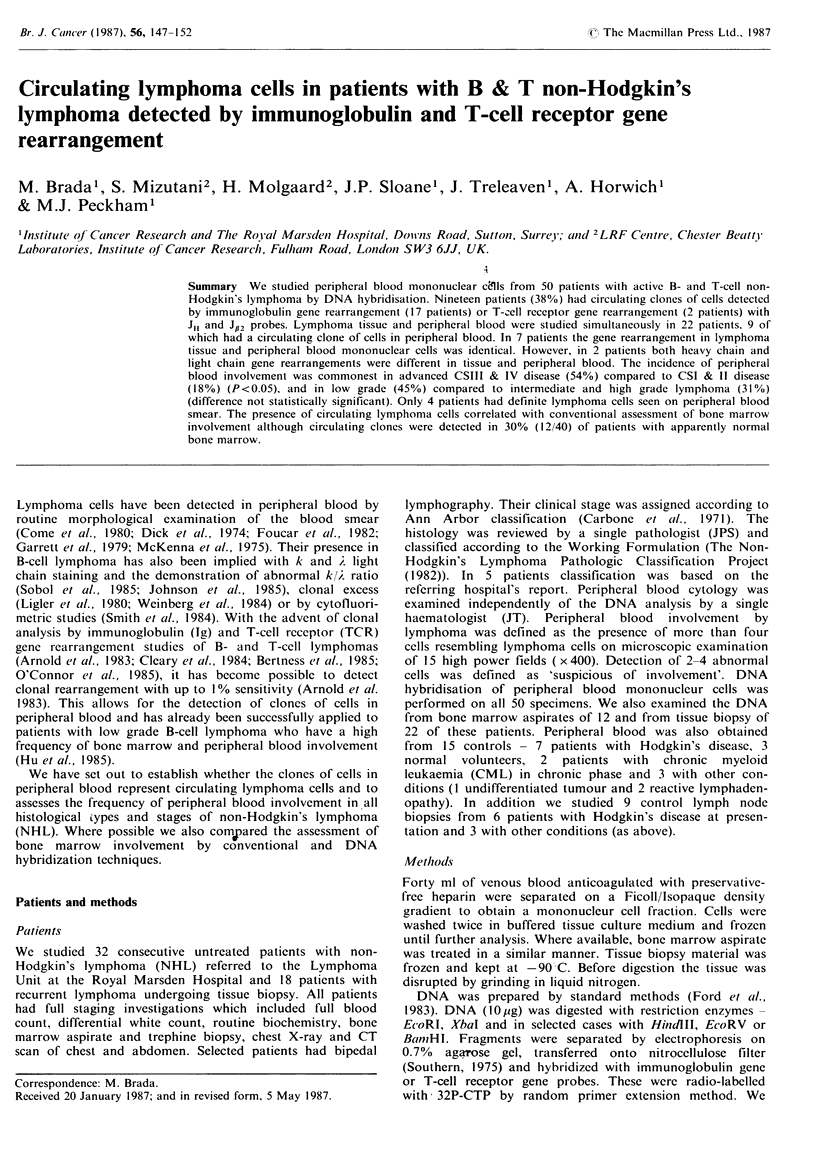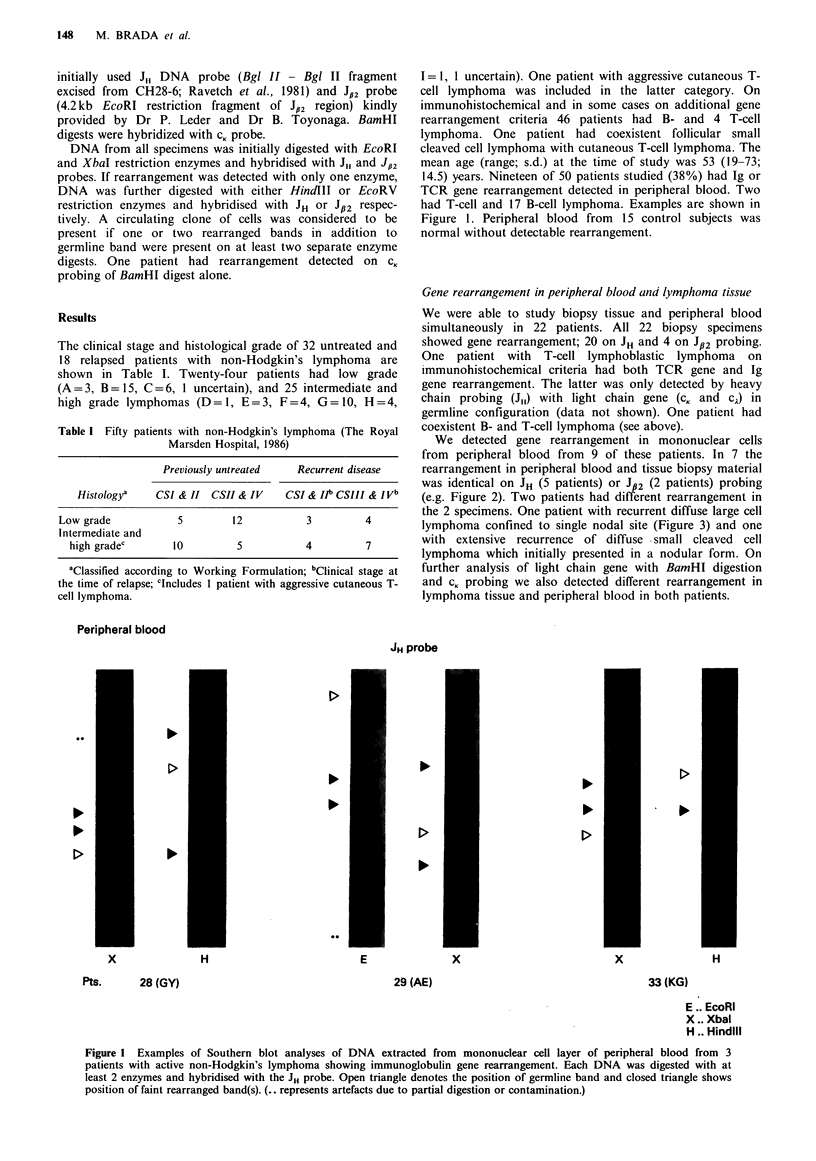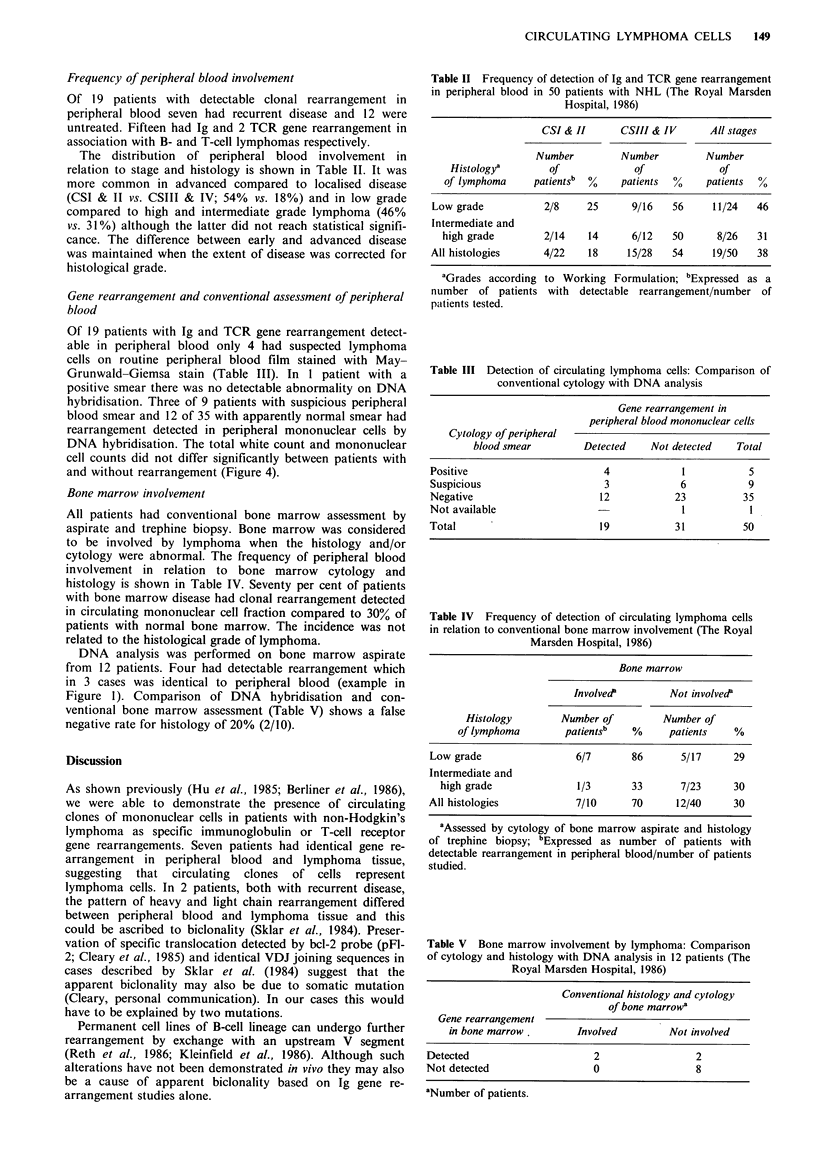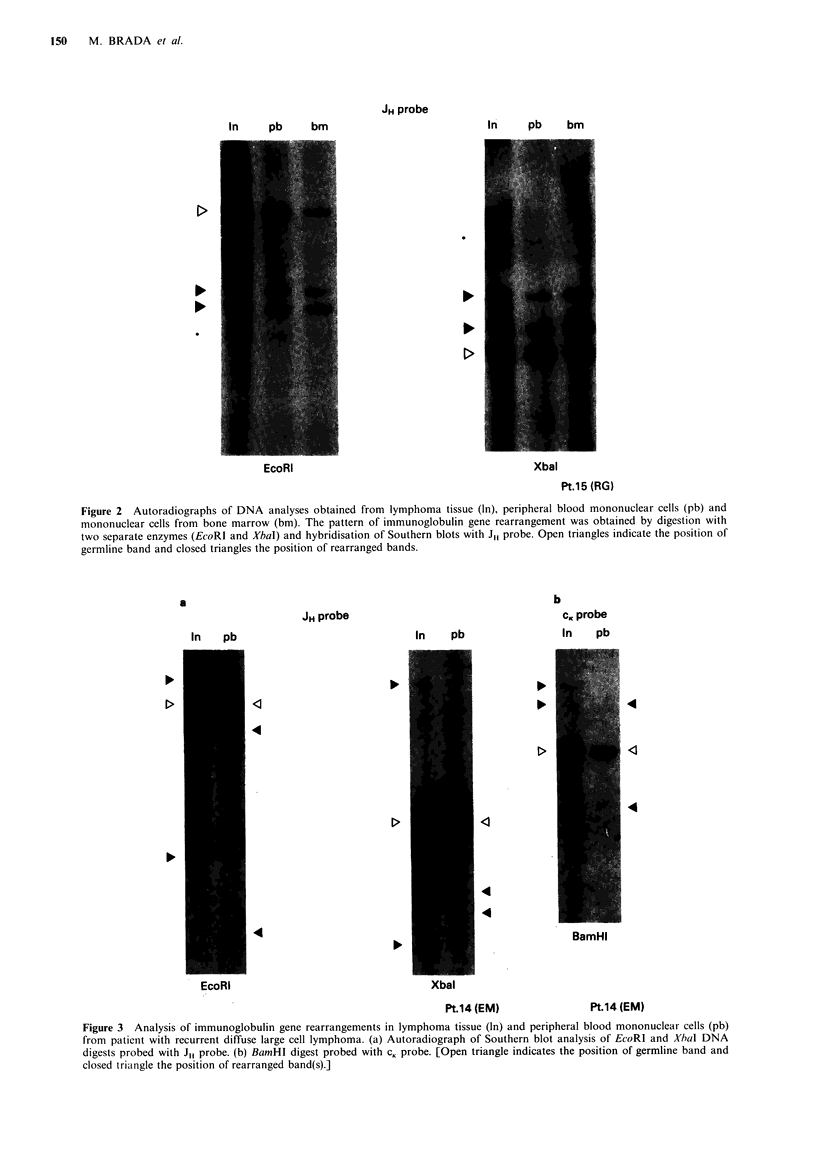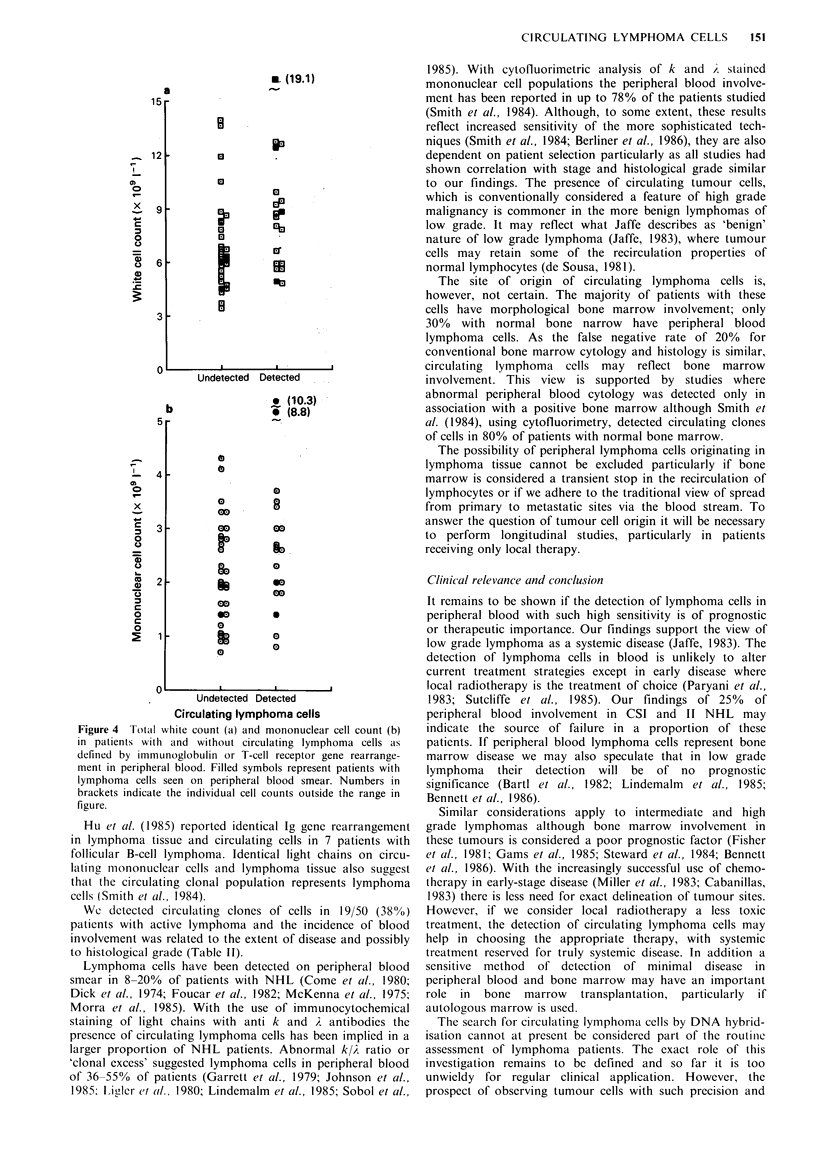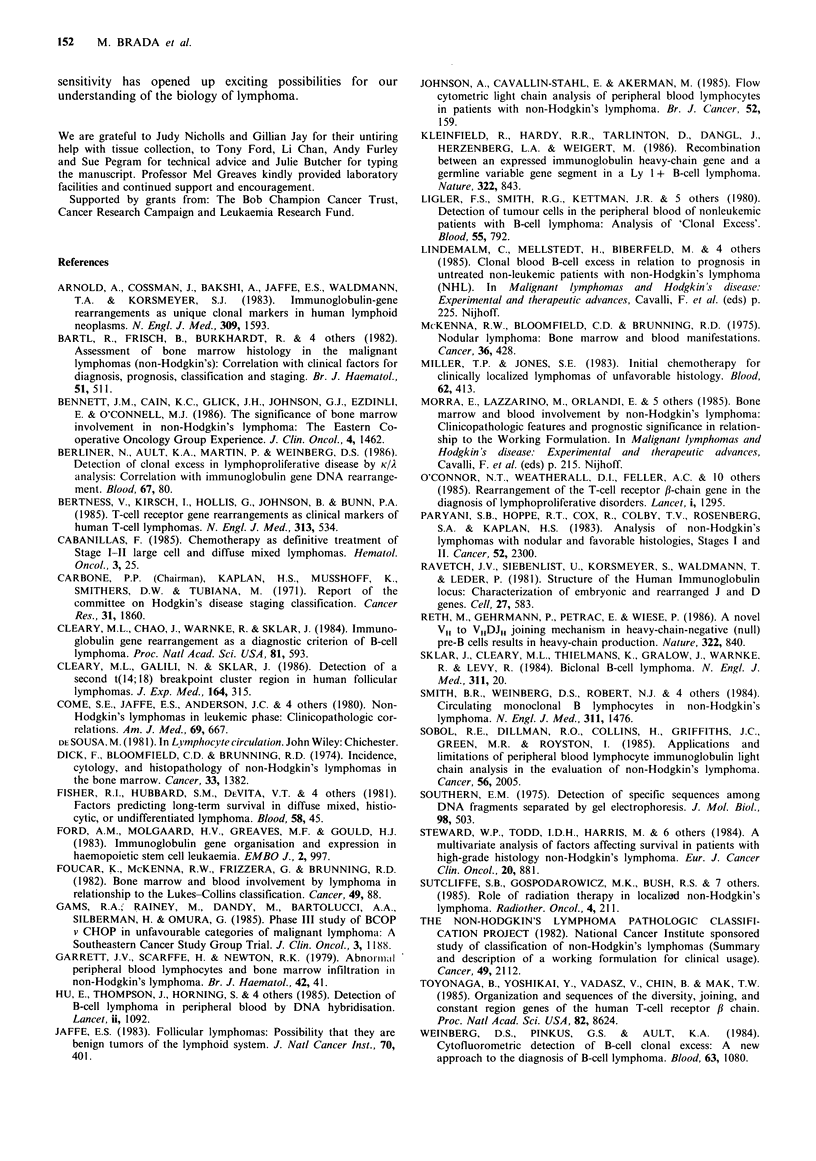# Circulating lymphoma cells in patients with B & T non-Hodgkin's lymphoma detected by immunoglobulin and T-cell receptor gene rearrangement.

**DOI:** 10.1038/bjc.1987.174

**Published:** 1987-08

**Authors:** M. Brada, S. Mizutani, H. Molgaard, J. P. Sloane, J. Treleaven, A. Horwich, M. J. Peckham

**Affiliations:** Institute of Cancer Research, Sutton, Surrey, UK.

## Abstract

**Images:**


Br. J. Cancer (1987), 56, 147-152 © The Macmillan Press Ltd., 1987

### Circulating lymphoma cells in patients with B & T non-Hodgkin's lymphoma detected by immunoglobulin and T-cell receptor gene rearrangement

M. Brada[1], S. Mizutani[2], H. Molgaard[2], J.P. Sloane[1], J. Treleaven[1], A. Horwich[1] & M.J. Peckham[1]

[1]*Institute of Cancer Research and The Royal Marsden Hospital, Downs Road, Sutton, Surrey; and* [2]*LRF Centre, Chester Beatty Laboratories, Institute of Cancer Research, Fulham Road, London SW3 6JJ, UK.*

**Summary** We studied peripheral blood mononuclear cells from 50 patients with active B- and T-cell non-Hodgkin's lymphoma by DNA hybridisation. Nineteen patients (38%) had circulating clones of cells detected by immunoglobulin gene rearrangement (17 patients) or T-cell receptor gene rearrangement (2 patients) with $J_H$ and $J_{\beta 2}$ probes. Lymphoma tissue and peripheral blood were studied simultaneously in 22 patients, 9 of which had a circulating clone of cells in peripheral blood. In 7 patients the gene rearrangement in lymphoma tissue and peripheral blood mononuclear cells was identical. However, in 2 patients both heavy chain and light chain gene rearrangements were different in tissue and peripheral blood. The incidence of peripheral blood involvement was commonest in advanced CSIII & IV disease (54%) compared to CSI & II disease (18%) ($P<0.05$), and in low grade (45%) compared to intermediate and high grade lymphoma (31%) (difference not statistically significant). Only 4 patients had definite lymphoma cells seen on peripheral blood smear. The presence of circulating lymphoma cells correlated with conventional assessment of bone marrow involvement although circulating clones were detected in 30% (12/40) of patients with apparently normal bone marrow.

Lymphoma cells have been detected in peripheral blood by routine morphological examination of the blood smear (Come *et al.*, 1980; Dick *et al.*, 1974; Foucar *et al.*, 1982; Garrett *et al.*, 1979; McKenna *et al.*, 1975). Their presence in B-cell lymphoma has also been implied with $k$ and $\lambda$ light chain staining and the demonstration of abnormal $k/\lambda$ ratio (Sobol *et al.*, 1985; Johnson *et al.*, 1985), clonal excess (Ligler *et al.*, 1980; Weinberg *et al.*, 1984) or by cytofluorimetric studies (Smith *et al.*, 1984). With the advent of clonal analysis by immunoglobulin (Ig) and T-cell receptor (TCR) gene rearrangement studies of B- and T-cell lymphomas (Arnold *et al.*, 1983; Cleary *et al.*, 1984; Bertness *et al.*, 1985; O'Connor *et al.*, 1985), it has become possible to detect clonal rearrangement with up to 1% sensitivity (Arnold *et al.* 1983). This allows for the detection of clones of cells in peripheral blood and has already been successfully applied to patients with low grade B-cell lymphoma who have a high frequency of bone marrow and peripheral blood involvement (Hu *et al.*, 1985).

We have set out to establish whether the clones of cells in peripheral blood represent circulating lymphoma cells and to assesses the frequency of peripheral blood involvement in all histological types and stages of non-Hodgkin's lymphoma (NHL). Where possible we also compared the assessment of bone marrow involvement by conventional and DNA hybridization techniques.

### Patients and methods

### *Patients*

We studied 32 consecutive untreated patients with non-Hodgkin's lymphoma (NHL) referred to the Lymphoma Unit at the Royal Marsden Hospital and 18 patients with recurrent lymphoma undergoing tissue biopsy. All patients had full staging investigations which included full blood count, differential white count, routine biochemistry, bone marrow aspirate and trephine biopsy, chest X-ray and CT scan of chest and abdomen. Selected patients had bipedal lymphography. Their clinical stage was assigned according to Ann Arbor classification (Carbone *et al.*, 1971). The histology was reviewed by a single pathologist (JPS) and classified according to the Working Formulation (The Non-Hodgkin's Lymphoma Pathologic Classification Project (1982)). In 5 patients classification was based on the referring hospital's report. Peripheral blood cytology was examined independently of the DNA analysis by a single haematologist (JT). Peripheral blood involvement by lymphoma was defined as the presence of more than four cells resembling lymphoma cells on microscopic examination of 15 high power fields (×400). Detection of 2–4 abnormal cells was defined as 'suspicious of involvement'. DNA hybridisation of peripheral blood mononucleur cells was performed on all 50 specimens. We also examined the DNA from bone marrow aspirates of 12 and from tissue biopsy of 22 of these patients. Peripheral blood was also obtained from 15 controls – 7 patients with Hodgkin's disease, 3 normal volunteers, 2 patients with chronic myeloid leukaemia (CML) in chronic phase and 3 with other conditions (1 undifferentiated tumour and 2 reactive lymphadenopathy). In addition we studied 9 control lymph node biopsies from 6 patients with Hodgkin's disease at presentation and 3 with other conditions (as above).

### *Methods*

Forty ml of venous blood anticoagulated with preservative-free heparin were separated on a Ficoll/Isopaque density gradient to obtain a mononucleur cell fraction. Cells were washed twice in buffered tissue culture medium and frozen until further analysis. Where available, bone marrow aspirate was treated in a similar manner. Tissue biopsy material was frozen and kept at −90°C. Before digestion the tissue was disrupted by grinding in liquid nitrogen.

DNA was prepared by standard methods (Ford *et al.*, 1983). DNA (10 μg) was digested with restriction enzymes – *Eco*RI, *Xba*I and in selected cases with *Hind*III, *Eco*RV or *Bam*HI. Fragments were separated by electrophoresis on 0.7% agarose gel, transferred onto nitrocellulose filter (Southern, 1975) and hybridized with immunoglobulin gene or T-cell receptor gene probes. These were radio-labelled with 32P-CTP by random primer extension method. We

Correspondence: M. Brada.
Received 20 January 1987; and in revised form, 5 May 1987.

initially used $J_H$ DNA probe (*Bgl II – Bgl* II fragment excised from CH28-6; Ravetch *et al.*, 1981) and $J_{\beta 2}$ probe (4.2 kb *Eco*RI restriction fragment of $J_{\beta 2}$ region) kindly provided by Dr P. Leder and Dr B. Toyonaga. *Bam*HI digests were hybridized with $c_\kappa$ probe.

DNA from all specimens was initially digested with *Eco*RI and *Xba*I restriction enzymes and hybridised with $J_H$ and $J_{\beta 2}$ probes. If rearrangement was detected with only one enzyme, DNA was further digested with either *Hind*III or *Eco*RV restriction enzymes and hybridised with $J_H$ or $J_{\beta 2}$ respectively. A circulating clone of cells was considered to be present if one or two rearranged bands in addition to germline band were present on at least two separate enzyme digests. One patient had rearrangement detected on $c_\kappa$ probing of *Bam*HI digest alone.

### Results

The clinical stage and histological grade of 32 untreated and 18 relapsed patients with non-Hodgkin's lymphoma are shown in Table I. Twenty-four patients had low grade (A = 3, B = 15, C = 6, 1 uncertain), and 25 intermediate and high grade lymphomas (D = 1, E = 3, F = 4, G = 10, H = 4, I = 1, 1 uncertain). One patient with aggressive cutaneous T-cell lymphoma was included in the latter category. On immunohistochemical and in some cases on additional gene rearrangement criteria 46 patients had B- and 4 T-cell lymphoma. One patient had coexistent follicular small cleaved cell lymphoma with cutaneous T-cell lymphoma. The mean age (range; s.d.) at the time of study was 53 (19–73; 14.5) years. Nineteen of 50 patients studied (38%) had Ig or TCR gene rearrangement detected in peripheral blood. Two had T-cell and 17 B-cell lymphoma. Examples are shown in Figure 1. Peripheral blood from 15 control subjects was normal without detectable rearrangement.

**Table I** Fifty patients with non-Hodgkin's lymphoma (The Royal Marsden Hospital, 1986)

| | *Previously untreated* | | *Recurrent disease* | |
|---|---|---|---|---|
| *Histology*[a] | *CSI & II* | *CSII & IV* | *CSI & II*[b] | *CSIII & IV*[b] |
| Low grade | 5 | 12 | 3 | 4 |
| Intermediate and high grade[c] | 10 | 5 | 4 | 7 |

[a]Classified according to Working Formulation; [b]Clinical stage at the time of relapse; [c]Includes 1 patient with aggressive cutaneous T-cell lymphoma.

### *Gene rearrangement in peripheral blood and lymphoma tissue*

We were able to study biopsy tissue and peripheral blood simultaneously in 22 patients. All 22 biopsy specimens showed gene rearrangement; 20 on $J_H$ and 4 on $J_{\beta 2}$ probing. One patient with T-cell lymphoblastic lymphoma on immunohistochemical criteria had both TCR gene and Ig gene rearrangement. The latter was only detected by heavy chain probing ($J_H$) with light chain gene ($c_\kappa$ and $c_\lambda$) in germline configuration (data not shown). One patient had coexistent B- and T-cell lymphoma (see above).

We detected gene rearrangement in mononuclear cells from peripheral blood from 9 of these patients. In 7 the rearrangement in peripheral blood and tissue biopsy material was identical on $J_H$ (5 patients) or $J_{\beta 2}$ (2 patients) probing (e.g. Figure 2). Two patients had different rearrangement in the 2 specimens. One patient with recurrent diffuse large cell lymphoma confined to single nodal site (Figure 3) and one with extensive recurrence of diffuse small cleaved cell lymphoma which initially presented in a nodular form. On further analysis of light chain gene with *Bam*HI digestion and $c_\kappa$ probing we also detected different rearrangement in lymphoma tissue and peripheral blood in both patients.

**Figure 1** Examples of Southern blot analyses of DNA extracted from mononuclear cell layer of peripheral blood from 3 patients with active non-Hodgkin's lymphoma showing immunoglobulin gene rearrangement. Each DNA was digested with at least 2 enzymes and hybridised with the $J_H$ probe. Open triangle denotes the position of germline band and closed triangle shows position of faint rearranged band(s). (.. represents artefacts due to partial digestion or contamination.)

*Frequency of peripheral blood involvement*

Of 19 patients with detectable clonal rearrangement in peripheral blood seven had recurrent disease and 12 were untreated. Fifteen had Ig and 2 TCR gene rearrangement in association with B- and T-cell lymphomas respectively.

The distribution of peripheral blood involvement in relation to stage and histology is shown in Table II. It was more common in advanced compared to localised disease (CSI & II *vs.* CSIII & IV; 54% *vs.* 18%) and in low grade compared to high and intermediate grade lymphoma (46% *vs.* 31%) although the latter did not reach statistical significance. The difference between early and advanced disease was maintained when the extent of disease was corrected for histological grade.

*Gene rearrangement and conventional assessment of peripheral blood*

Of 19 patients with Ig and TCR gene rearrangement detectable in peripheral blood only 4 had suspected lymphoma cells on routine peripheral blood film stained with May-Grunwald-Giemsa stain (Table III). In 1 patient with a positive smear there was no detectable abnormality on DNA hybridisation. Three of 9 patients with suspicious peripheral blood smear and 12 of 35 with apparently normal smear had rearrangement detected in peripheral mononuclear cells by DNA hybridisation. The total white count and mononuclear cell counts did not differ significantly between patients with and without rearrangement (Figure 4).

*Bone marrow involvement*

All patients had conventional bone marrow assessment by aspirate and trephine biopsy. Bone marrow was considered to be involved by lymphoma when the histology and/or cytology were abnormal. The frequency of peripheral blood involvement in relation to bone marrow cytology and histology is shown in Table IV. Seventy per cent of patients with bone marrow disease had clonal rearrangement detected in circulating mononuclear cell fraction compared to 30% of patients with normal bone marrow. The incidence was not related to the histological grade of lymphoma.

DNA analysis was performed on bone marrow aspirate from 12 patients. Four had detectable rearrangement which in 3 cases was identical to peripheral blood (example in Figure 1). Comparison of DNA hybridisation and conventional bone marrow assessment (Table V) shows a false negative rate for histology of 20% (2/10).

**Table II** Frequency of detection of Ig and TCR gene rearrangement in peripheral blood in 50 patients with NHL (The Royal Marsden Hospital, 1986)

| Histology[a] of lymphoma | CSI & II Number of patients[b] | % | CSIII & IV Number of patients | % | All stages Number of patients | % |
|---|---|---|---|---|---|---|
| Low grade | 2/8 | 25 | 9/16 | 56 | 11/24 | 46 |
| Intermediate and high grade | 2/14 | 14 | 6/12 | 50 | 8/26 | 31 |
| All histologies | 4/22 | 18 | 15/28 | 54 | 19/50 | 38 |

[a]Grades according to Working Formulation; [b]Expressed as a number of patients with detectable rearrangement/number of patients tested.

**Table III** Detection of circulating lymphoma cells: Comparison of conventional cytology with DNA analysis

| Cytology of peripheral blood smear | Gene rearrangement in peripheral blood mononuclear cells: Detected | Not detected | Total |
|---|---|---|---|
| Positive | 4 | 1 | 5 |
| Suspicious | 3 | 6 | 9 |
| Negative | 12 | 23 | 35 |
| Not available | — | 1 | 1 |
| Total | 19 | 31 | 50 |

**Table IV** Frequency of detection of circulating lymphoma cells in relation to conventional bone marrow involvement (The Royal Marsden Hospital, 1986)

| Histology of lymphoma | Bone marrow Involved[a] Number of patients[b] | % | Not involved[a] Number of patients | % |
|---|---|---|---|---|
| Low grade | 6/7 | 86 | 5/17 | 29 |
| Intermediate and high grade | 1/3 | 33 | 7/23 | 30 |
| All histologies | 7/10 | 70 | 12/40 | 30 |

[a]Assessed by cytology of bone marrow aspirate and histology of trephine biopsy; [b]Expressed as number of patients with detectable rearrangement in peripheral blood/number of patients studied.

**Table V** Bone marrow involvement by lymphoma: Comparison of cytology and histology with DNA analysis in 12 patients (The Royal Marsden Hospital, 1986)

| Gene rearrangement in bone marrow | Conventional histology and cytology of bone marrow[a]: Involved | Not involved |
|---|---|---|
| Detected | 2 | 2 |
| Not detected | 0 | 8 |

[a]Number of patients.

### Discussion

As shown previously (Hu *et al.*, 1985; Berliner *et al.*, 1986), we were able to demonstrate the presence of circulating clones of mononuclear cells in patients with non-Hodgkin's lymphoma as specific immunoglobulin or T-cell receptor gene rearrangements. Seven patients had identical gene rearrangement in peripheral blood and lymphoma tissue, suggesting that circulating clones of cells represent lymphoma cells. In 2 patients, both with recurrent disease, the pattern of heavy and light chain rearrangement differed between peripheral blood and lymphoma tissue and this could be ascribed to biclonality (Sklar *et al.*, 1984). Preservation of specific translocation detected by bcl-2 probe (pFl-2; Cleary *et al.*, 1985) and identical VDJ joining sequences in cases described by Sklar *et al.* (1984) suggest that the apparent biclonality may also be due to somatic mutation (Cleary, personal communication). In our cases this would have to be explained by two mutations.

Permanent cell lines of B-cell lineage can undergo further rearrangement by exchange with an upstream V segment (Reth *et al.*, 1986; Kleinfield *et al.*, 1986). Although such alterations have not been demonstrated *in vivo* they may also be a cause of apparent biclonality based on Ig gene rearrangement studies alone.

**Figure 2** Autoradiographs of DNA analyses obtained from lymphoma tissue (ln), peripheral blood mononuclear cells (pb) and mononuclear cells from bone marrow (bm). The pattern of immunoglobulin gene rearrangement was obtained by digestion with two separate enzymes (*Eco*RI and *Xba*I) and hybridisation of Southern blots with $J_H$ probe. Open triangles indicate the position of germline band and closed triangles the position of rearranged bands.

**Figure 3** Analysis of immunoglobulin gene rearrangements in lymphoma tissue (ln) and peripheral blood mononuclear cells (pb) from patient with recurrent diffuse large cell lymphoma. (a) Autoradiograph of Southern blot analysis of *Eco*RI and *Xba*I DNA digests probed with $J_H$ probe. (b) *Bam*HI digest probed with $c_\kappa$ probe. [Open triangle indicates the position of germline band and closed triangle the position of rearranged band(s).]

**Figure 4** Total white count (a) and mononuclear cell count (b) in patients with and without circulating lymphoma cells as defined by immunoglobulin or T-cell receptor gene rearrangement in peripheral blood. Filled symbols represent patients with lymphoma cells seen on peripheral blood smear. Numbers in brackets indicate the individual cell counts outside the range in figure.

Hu *et al.* (1985) reported identical Ig gene rearrangement in lymphoma tissue and circulating cells in 7 patients with follicular B-cell lymphoma. Identical light chains on circulating mononuclear cells and lymphoma tissue also suggest that the circulating clonal population represents lymphoma cells (Smith *et al.*, 1984).

We detected circulating clones of cells in 19/50 (38%) patients with active lymphoma and the incidence of blood involvement was related to the extent of disease and possibly to histological grade (Table II).

Lymphoma cells have been detected on peripheral blood smear in 8–20% of patients with NHL (Come *et al.*, 1980; Dick *et al.*, 1974; Foucar *et al.*, 1982; McKenna *et al.*, 1975; Morra *et al.*, 1985). With the use of immunocytochemical staining of light chains with anti $k$ and $\lambda$ antibodies the presence of circulating lymphoma cells has been implied in a larger proportion of NHL patients. Abnormal $k/\lambda$ ratio or 'clonal excess' suggested lymphoma cells in peripheral blood of 36–55% of patients (Garrett *et al.*, 1979; Johnson *et al.*, 1985; Ligler *et al.*, 1980; Lindemalm *et al.*, 1985; Sobol *et al.*, 1985). With cytofluorimetric analysis of $k$ and $\lambda$ stained mononuclear cell populations the peripheral blood involvement has been reported in up to 78% of the patients studied (Smith *et al.*, 1984). Although, to some extent, these results reflect increased sensitivity of the more sophisticated techniques (Smith *et al.*, 1984; Berliner *et al.*, 1986), they are also dependent on patient selection particularly as all studies had shown correlation with stage and histological grade similar to our findings. The presence of circulating tumour cells, which is conventionally considered a feature of high grade malignancy is commoner in the more benign lymphomas of low grade. It may reflect what Jaffe describes as 'benign' nature of low grade lymphoma (Jaffe, 1983), where tumour cells may retain some of the recirculation properties of normal lymphocytes (de Sousa, 1981).

The site of origin of circulating lymphoma cells is, however, not certain. The majority of patients with these cells have morphological bone marrow involvement; only 30% with normal bone narrow have peripheral blood lymphoma cells. As the false negative rate of 20% for conventional bone marrow cytology and histology is similar, circulating lymphoma cells may reflect bone marrow involvement. This view is supported by studies where abnormal peripheral blood cytology was detected only in association with a positive bone marrow although Smith *et al.* (1984), using cytofluorimetry, detected circulating clones of cells in 80% of patients with normal bone marrow.

The possibility of peripheral lymphoma cells originating in lymphoma tissue cannot be excluded particularly if bone marrow is considered a transient stop in the recirculation of lymphocytes or if we adhere to the traditional view of spread from primary to metastatic sites via the blood stream. To answer the question of tumour cell origin it will be necessary to perform longitudinal studies, particularly in patients receiving only local therapy.

### *Clinical relevance and conclusion*

It remains to be shown if the detection of lymphoma cells in peripheral blood with such high sensitivity is of prognostic or therapeutic importance. Our findings support the view of low grade lymphoma as a systemic disease (Jaffe, 1983). The detection of lymphoma cells in blood is unlikely to alter current treatment strategies except in early disease where local radiotherapy is the treatment of choice (Paryani *et al.*, 1983; Sutcliffe *et al.*, 1985). Our findings of 25% of peripheral blood involvement in CSI and II NHL may indicate the source of failure in a proportion of these patients. If peripheral blood lymphoma cells represent bone marrow disease we may also speculate that in low grade lymphoma their detection will be of no prognostic significance (Bartl *et al.*, 1982; Lindemalm *et al.*, 1985; Bennett *et al.*, 1986).

Similar considerations apply to intermediate and high grade lymphomas although bone marrow involvement in these tumours is considered a poor prognostic factor (Fisher *et al.*, 1981; Gams *et al.*, 1985; Steward *et al.*, 1984; Bennett *et al.*, 1986). With the increasingly successful use of chemotherapy in early-stage disease (Miller *et al.*, 1983; Cabanillas, 1983) there is less need for exact delineation of tumour sites. However, if we consider local radiotherapy a less toxic treatment, the detection of circulating lymphoma cells may help in choosing the appropriate therapy, with systemic treatment reserved for truly systemic disease. In addition a sensitive method of detection of minimal disease in peripheral blood and bone marrow may have an important role in bone marrow transplantation, particularly if autologous marrow is used.

The search for circulating lymphoma cells by DNA hybridisation cannot at present be considered part of the routine assessment of lymphoma patients. The exact role of this investigation remains to be defined and so far it is too unwieldy for regular clinical application. However, the prospect of observing tumour cells with such precision and sensitivity has opened up exciting possibilities for our understanding of the biology of lymphoma.

We are grateful to Judy Nicholls and Gillian Jay for their untiring help with tissue collection, to Tony Ford, Li Chan, Andy Furley and Sue Pegram for technical advice and Julie Butcher for typing the manuscript. Professor Mel Greaves kindly provided laboratory facilities and continued support and encouragement.

Supported by grants from: The Bob Champion Cancer Trust, Cancer Research Campaign and Leukaemia Research Fund.

### References

ARNOLD, A., COSSMAN, J., BAKSHI, A., JAFFE, E.S., WALDMANN, T.A. & KORSMEYER, S.J. (1983). Immunoglobulin-gene rearrangements as unique clonal markers in human lymphoid neoplasms. *N. Engl. J. Med.*, **309**, 1593.

BARTL, R., FRISCH, B., BURKHARDT, R. & 4 others (1982). Assessment of bone marrow histology in the malignant lymphomas (non-Hodgkin's): Correlation with clinical factors for diagnosis, prognosis, classification and staging. *Br. J. Haematol.*, **51**, 511.

BENNETT, J.M., CAIN, K.C., GLICK, J.H., JOHNSON, G.J., EZDINLI, E. & O'CONNELL, M.J. (1986). The significance of bone marrow involvement in non-Hodgkin's lymphoma: The Eastern Co-operative Oncology Group Experience. *J. Clin. Oncol.*, **4**, 1462.

BERLINER, N., AULT, K.A., MARTIN, P. & WEINBERG, D.S. (1986). Detection of clonal excess in lymphoproliferative disease by $\kappa/\lambda$ analysis: Correlation with immunoglobulin gene DNA rearrangement. *Blood*, **67**, 80.

BERTNESS, V., KIRSCH, I., HOLLIS, G., JOHNSON, B. & BUNN, P.A. (1985). T-cell receptor gene rearrangements as clinical markers of human T-cell lymphomas. *N. Engl. J. Med.*, **313**, 534.

CABANILLAS, F. (1985). Chemotherapy as definitive treatment of Stage I–II large cell and diffuse mixed lymphomas. *Hematol. Oncol.*, **3**, 25.

CARBONE, P.P. (Chairman), KAPLAN, H.S., MUSSHOFF, K., SMITHERS, D.W. & TUBIANA, M. (1971). Report of the committee on Hodgkin's disease staging classification. *Cancer Res.*, **31**, 1860.

CLEARY, M.L., CHAO, J., WARNKE, R. & SKLAR, J. (1984). Immunoglobulin gene rearrangement as a diagnostic criterion of B-cell lymphoma. *Proc. Natl Acad. Sci. USA*, **81**, 593.

CLEARY, M.L., GALILI, N. & SKLAR, J. (1986). Detection of a second t(14;18) breakpoint cluster region in human follicular lymphomas. *J. Exp. Med.*, **164**, 315.

COME, S.E., JAFFE, E.S., ANDERSON, J.C. & 4 others (1980). Non-Hodgkin's lymphomas in leukemic phase: Clinicopathologic correlations. *Am. J. Med.*, **69**, 667.

DE SOUSA, M. (1981). In *Lymphocyte circulation*. John Wiley: Chichester.

DICK, F., BLOOMFIELD, C.D. & BRUNNING, R.D. (1974). Incidence, cytology, and histopathology of non-Hodgkin's lymphomas in the bone marrow. *Cancer*, **33**, 1382.

FISHER, R.I., HUBBARD, S.M., DEVITA, V.T. & 4 others (1981). Factors predicting long-term survival in diffuse mixed, histiocytic, or undifferentiated lymphoma. *Blood*, **58**, 45.

FORD, A.M., MOLGAARD, H.V., GREAVES, M.F. & GOULD, H.J. (1983). Immunoglobulin gene organisation and expression in haemopoietic stem cell leukaemia. *EMBO J.*, **2**, 997.

FOUCAR, K., McKENNA, R.W., FRIZZERA, G. & BRUNNING, R.D. (1982). Bone marrow and blood involvement by lymphoma in relationship to the Lukes–Collins classification. *Cancer*, **49**, 88.

GAMS, R.A., RAINEY, M., DANDY, M., BARTOLUCCI, A.A., SILBERMAN, H. & OMURA, G. (1985). Phase III study of BCOP *v* CHOP in unfavourable categories of malignant lymphoma: A Southeastern Cancer Study Group Trial. *J. Clin. Oncol.*, **3**, 1188.

GARRETT, J.V., SCARFFE, H. & NEWTON, R.K. (1979). Abnormal peripheral blood lymphocytes and bone marrow infiltration in non-Hodgkin's lymphoma. *Br. J. Haematol.*, **42**, 41.

HU, E., THOMPSON, J., HORNING, S. & 4 others (1985). Detection of B-cell lymphoma in peripheral blood by DNA hybridisation. *Lancet*, **ii**, 1092.

JAFFE, E.S. (1983). Follicular lymphomas: Possibility that they are benign tumors of the lymphoid system. *J. Natl Cancer Inst.*, **70**, 401.

JOHNSON, A., CAVALLIN-STAHL, E. & AKERMAN, M. (1985). Flow cytometric light chain analysis of peripheral blood lymphocytes in patients with non-Hodgkin's lymphoma. *Br. J. Cancer*, **52**, 159.

KLEINFIELD, R., HARDY, R.R., TARLINTON, D., DANGL, J., HERZENBERG, L.A. & WEIGERT, M. (1986). Recombination between an expressed immunoglobulin heavy-chain gene and a germline variable gene segment in a Ly 1+ B-cell lymphoma. *Nature*, **322**, 843.

LIGLER, F.S., SMITH, R.G., KETTMAN, J.R. & 5 others (1980). Detection of tumour cells in the peripheral blood of nonleukemic patients with B-cell lymphoma: Analysis of 'Clonal Excess'. *Blood*, **55**, 792.

LINDEMALM, C., MELLSTEDT, H., BIBERFELD, M. & 4 others (1985). Clonal blood B-cell excess in relation to prognosis in untreated non-leukemic patients with non-Hodgkin's lymphoma (NHL). In *Malignant lymphomas and Hodgkin's disease: Experimental and therapeutic advances*, Cavalli, F. *et al.* (eds) p. 225. Nijhoff.

McKENNA, R.W., BLOOMFIELD, C.D. & BRUNNING, R.D. (1975). Nodular lymphoma: Bone marrow and blood manifestations. *Cancer*, **36**, 428.

MILLER, T.P. & JONES, S.E. (1983). Initial chemotherapy for clinically localized lymphomas of unfavorable histology. *Blood*, **62**, 413.

MORRA, E., LAZZARINO, M., ORLANDI, E. & 5 others (1985). Bone marrow and blood involvement by non-Hodgkin's lymphoma: Clinicopathologic features and prognostic significance in relationship to the Working Formulation. In *Malignant lymphomas and Hodgkin's disease: Experimental and therapeutic advances*, Cavalli, F. *et al.* (eds) p. 215. Nijhoff.

O'CONNOR, N.T., WEATHERALL, D.I., FELLER, A.C. & 10 others (1985). Rearrangement of the T-cell receptor $\beta$-chain gene in the diagnosis of lymphoproliferative disorders. *Lancet*, **i**, 1295.

PARYANI, S.B., HOPPE, R.T., COX, R., COLBY, T.V., ROSENBERG, S.A. & KAPLAN, H.S. (1983). Analysis of non-Hodgkin's lymphomas with nodular and favorable histologies, Stages I and II. *Cancer*, **52**, 2300.

RAVETCH, J.V., SIEBENLIST, U., KORSMEYER, S., WALDMANN, T. & LEDER, P. (1981). Structure of the Human Immunoglobulin locus: Characterization of embryonic and rearranged J and D genes. *Cell*, **27**, 583.

RETH, M., GEHRMANN, P., PETRAC, E. & WIESE, P. (1986). A novel $V_H$ to $V_HDJ_H$ joining mechanism in heavy-chain-negative (null) pre-B cells results in heavy-chain production. *Nature*, **322**, 840.

SKLAR, J., CLEARY, M.L., THIELMANS, K., GRALOW, J., WARNKE, R. & LEVY, R. (1984). Biclonal B-cell lymphoma. *N. Engl. J. Med.*, **311**, 20.

SMITH, B.R., WEINBERG, D.S., ROBERT, N.J. & 4 others (1984). Circulating monoclonal B lymphocytes in non-Hodgkin's lymphoma. *N. Engl. J. Med.*, **311**, 1476.

SOBOL, R.E., DILLMAN, R.O., COLLINS, H., GRIFFITHS, J.C., GREEN, M.R. & ROYSTON, I. (1985). Applications and limitations of peripheral blood lymphocyte immunoglobulin light chain analysis in the evaluation of non-Hodgkin's lymphoma. *Cancer*, **56**, 2005.

SOUTHERN, E.M. (1975). Detection of specific sequences among DNA fragments separated by gel electrophoresis. *J. Mol. Biol.*, **98**, 503.

STEWARD, W.P., TODD, I.D.H., HARRIS, M. & 6 others (1984). A multivariate analysis of factors affecting survival in patients with high-grade histology non-Hodgkin's lymphoma. *Eur. J. Cancer Clin. Oncol.*, **20**, 881.

SUTCLIFFE, S.B., GOSPODAROWICZ, M.K., BUSH, R.S. & 7 others. (1985). Role of radiation therapy in localized non-Hodgkin's lymphoma. *Radiother. Oncol.*, **4**, 211.

THE NON-HODGKIN'S LYMPHOMA PATHOLOGIC CLASSIFICATION PROJECT (1982). National Cancer Institute sponsored study of classification of non-Hodgkin's lymphomas (Summary and description of a working formulation for clinical usage). *Cancer*, **49**, 2112.

TOYONAGA, B., YOSHIKAI, Y., VADASZ, V., CHIN, B. & MAK, T.W. (1985). Organization and sequences of the diversity, joining, and constant region genes of the human T-cell receptor $\beta$ chain. *Proc. Natl Acad. Sci. USA*, **82**, 8624.

WEINBERG, D.S., PINKUS, G.S. & AULT, K.A. (1984). Cytofluorometric detection of B-cell clonal excess: A new approach to the diagnosis of B-cell lymphoma. *Blood*, **63**, 1080.